# Genome Sequence of *Bacillus cereus* Strain A1, an Efficient Starch-Utilizing Producer of Hydrogen

**DOI:** 10.1128/genomeA.00494-14

**Published:** 2014-05-29

**Authors:** Ting Zhang, Meidan Bao, Yu Wang, Haijia Su, Tianwei Tan

**Affiliations:** Beijing Key Laboratory of Bioprocess, Beijing University of Chemical Technology, Beijing, China

## Abstract

*Bacillus cereus* strain A1 is a newly isolated hydrogen producer capable of utilizing bioresources and biowaste, such as starch and starch wastewater. Here, we present a 5.67-Mb assembly of the genome sequence of strain A1, which may provide insights into the molecular mechanism of hydrogen production from bioresources and biowaste.

## GENOME ANNOUNCEMENT

Hydrogen (H_2_) has been considered one of the potential energy carriers for the future due to its advantages over classical hydrocarbon fuels, such as high efficiency, recyclability, and nonpolluting nature (1, 2). Various types of biomass have been used for H_2_ production via biological processes (1, 3). Starch is rich in carbohydrates and its hydrolyzed sugar can be easily converted to H_2_ by bacteria (4). Corn and cassava starches are abundantly available in China, as well as the wastewater from the starch manufacturing process (4, 5). Although large numbers of organisms, in both pure and mixed cultures, have been widely studied for H_2_ production from biomass (1, 6–8), H_2_ producers using unhydrolyzed starch directly are rare (4, 9).

*Bacillus cereus* strain A1 is a facultative anaerobe isolated from the activated sludge of an anaerobic digestion reactor. Strain A1 hydrolyzes starch by secreting amylase and fermenting glucose into H_2_ (4, 9). In addition to glucose and unhydrolyzed starch, strain A1 also utilizes biowastes such as starch wastewater to produce H_2_ (data not shown in detail). The end products are acetate and ethanol (4). Interestingly, when *B. cereus* strain A1 was cocultured with another H_2_ producer, *Brevumdimonas naejangsanensis* strain B1, a high efficiency of H_2_ production was obtained and the end products were mainly butyrate and acetate (4). *B. cereus* strain A1 quenches O_2_ and provides substrates for H_2_ production in a mixed culture, showing promising potential applications in the biohydrogen industry (1, 4).

Here, we present the draft genome sequence of *B. cereus* strain A1, obtained by using the Illumina HiSeq 2000 system, which was performed by the Chinese National Human Genome Center, Shanghai, China, with a paired-end library. The reads were assembled into 58 contigs by using VELVET (10). The genome annotation was performed by the RAST server (11). The G+C content was calculated by using the genome sequence.

The draft genome sequence of strain A1 comprises 5,667,342 bases, with a G+C content of 34.9%. The genome was predicted to contain 5,699 coding sequences (CDSs) together with 112 RNAs. According to the annotation, we have predicted CDSs related to a pentose metabolite pathway. The transketolase/transaldolase pathway, instead of phosphoketolase, was predicted in the genome, implying that strain A1 might utilize pentose more efficiently. Five CDSs responsible for sucrose utilization, 10 CDSs for fructose utilization, and 16 CDSs for glycerol utilization, in addition to alpha-amylase-encoding-genes, were also annotated, suggesting that strain A1 may have a wide substrate spectrum. Moreover, there are 12 CDSs related to acetoin and butanediol metabolism. Several CDSs responsible for organic acid formation were annotated, including formate, acetate, lactate, and butyrate. Further investigation of the pathways and key genes may help to reveal the mechanism of H_2_ production in strain A1 and to improve the production efficiency.

### Nucleotide sequence accession numbers. 

The whole-genome shotgun project has been deposited at DDBJ/EMBL/GenBank under the accession number JHOG00000000. The version described in this paper is the first version, JHOG01000000.
